# Effect of gait types and external weight carrying strategies on the femoral neck strains during stair descent

**DOI:** 10.1371/journal.pone.0294181

**Published:** 2023-11-21

**Authors:** Chen Deng, Jason C. Gillette, Timothy R. Derrick

**Affiliations:** 1 Division of Sport Biomechanics, School of Sport Science, Beijing Sport University, Beijing, P.R China; 2 Department of Kinesiology, Iowa State University, Ames, IA, United States of America; Kennedy Krieger Institute/Johns Hopkins University School of Medicine, UNITED STATES

## Abstract

Gait and weight carrying method may change the femoral neck load during stair descent. Applying specific gait and weight carrying methods may reduce the femoral neck load during stair descent, which may reduce hip pain, hip pain related falls and fall related fractures for the older population. The purpose of this study was to test the effect of different gait types (step-over-step v.s. step-by-step) and external weight carrying strategies (ipsilateral v.s. contralateral side) on the femoral neck load, discover which method could reduce the femoral neck load effectively. Seventeen healthy adults from 50 to 70 yrs old were recruited. The kinematic and kinetic analysis, musculoskeletal modelling method were used to estimate the joint and muscle loads for the lower extremities. Finite element analysis was used with the femur model to calculate the femoral neck strains during stair descent with different gait types and weight carrying strategies. The compressive strains were reduced for step-by-step gait method than step-over-step (p<0.015, 12.3–17.4% decrease of strains), the tensile strains were significantly increased for the trailing leg of step-by-step than the leading leg (p<0.001, 24.7% increase of strains). Contralateral weight carrying increased compressive and tensile strains than ipsilateral (p<0.001, 9.9–24.5% increase of strains) in most conditions. Applying step-by-step method and avoiding contralateral side weight carrying could be effective to reduce femoral neck strains. These outcomes could be helpful for the older population to reduce the risks of hip pain, femoral neck pain or pain related falls and fractures.

## Introduction

Fractures at the hip and the proximal femur, especially femoral neck fractures, are among the most serious injuries in the world which could cause serious health issues. These injuries could reduce the quality of life, and result in life-threatening issues and heavy economic burden for the older population and their families [1–6]. Due to the increasing of cases and issues with these fractures for the growing aging population [6, 7], studying the mechanism of femoral neck fractures is necessary. For the older population, hip joint pain and falls are more reported during stair walk, especially during stair descent. It is shown that one third of older population experienced falls in the life time, the risks of serious injuries (fractures, concussions etc.) due to falling were about 20–30% for the older population [8–10]. Since the external loading at the proximal femur region (especially at the femoral neck) could be one major risk factor for the femoral neck fractures, hip/femoral pain and pain related falls [10–15], the analysis of the load environment for the femoral neck could be helpful to minimize further damage to an injured site or develop preventative measures to reduce mechanical load on the femoral neck [16].

Previous studies utilized different methods to estimate joint load or femoral load for the proximal thigh region. Analysis of the proximal femur load helps understand the mechanisms of failure. Using instrumented hip prostheses, the load environment at the proximal femur for stair walk [17–21] had been measured. Due to the small sample size and atypical subjects, the results of these measurements could not be applied to the healthy population. The development of indirect measurements allowed inverse dynamics and rigid body models to estimate hip joint moments and reaction forces for stair walk [22, 23]. Greater hip moments were found for stair descent than ascent. But the effect of co-contracting muscles, the geometry and material properties of the bone were not considered in the analysis. The 2-dimensional simplified model was utilized for stair ascent and descent to estimate normal stress on the certain cross-sections of the femoral neck [24, 25]. But this simplified model could not estimate the 3-dimensional strains to represent the deformation of the structure.

The finite element analysis method (FEM) could provide the estimation of stresses or strains for the bone. In this analysis, the bone models with material property and geometry (from CT scans etc.), the musculoskeletal model and force estimation methods were used to get bone load estimation [26–28]. For vivo testing, strains on the femur model could predict how much micro-deformation could be acting on the femur [29–32], which revealed the load environment of the bone and analyzed the bone load which could be responsible for injuries for many daily activities: Anderson et al., [29] compared strains at the proximal femur between the older and young adults’ models during normal walking, which showed that strains for older adults were generally similar with or greater than young adults; Edwards et al., [30] used the methods of static optimization and forward dynamics to analyze femoral strains during normal walking, which showed a good agreement with the results from the strain gage measurements; Kersh et al., [31] and Martelli et al., [32] tested more activities (jumping, stair walking, and normal walking etc.), which showed greater strains during stair ascent/descent than normal walking at the superior and anterior aspect of the femoral neck.

According to the previous work, greater stresses/strains were found during stair descent than stair ascent for the femoral neck region [24, 25], which may result in more hip/femoral pain and pain related falls [10–15]. More protective strategies (change gait types, weight carrying strategies etc.) should be developed to reduce femoral neck load during stair descent, which could reduce the risk of hip/femoral pain and pain related falls [12–15], and further reduce the risk of fractures for the proximal femur. The effect of these possible protective strategies were not analyzed previously and this study would examine if the change of the gait types or external weight carrying strategies could reduce femoral neck strains.

In this study, maximal compressive (defined as the 3^rd^ principal strain) and tensile (defined as the 1^st^ principal strain) strains were tested at the femoral neck region using different gait types (step-over-step, step-by-step) and external weight carrying strategies (no load, 10% of body weight at the ipsilateral side, 10% at the contralateral side) during stair descent. The purposes of this study were to 1) test the effect of gait types, weight carrying strategies on the femoral neck strains; 2) find the preventative methods to reduce femoral neck strains. It was assumed that 1) the gait type of step-by-step would significantly reduce the femoral neck strains than step-over-step; and 2) the contralateral weight carrying would increase femoral neck strains than the ipsilateral strategy.

## Methods

Seventeen adults from 50 to 70 yrs old volunteered to participate (male: 8; females: 9; age: 57.1±5.9 yrs, height: 1.70±0.06 m, weight: 69±11.0 kg, without lower limb injuries). Before participation, all the participants signed the written informed consent document that had been approved by the Iowa State University Human Subjects Review Board (ISU IRB # 17–296).

**Fig 1:**
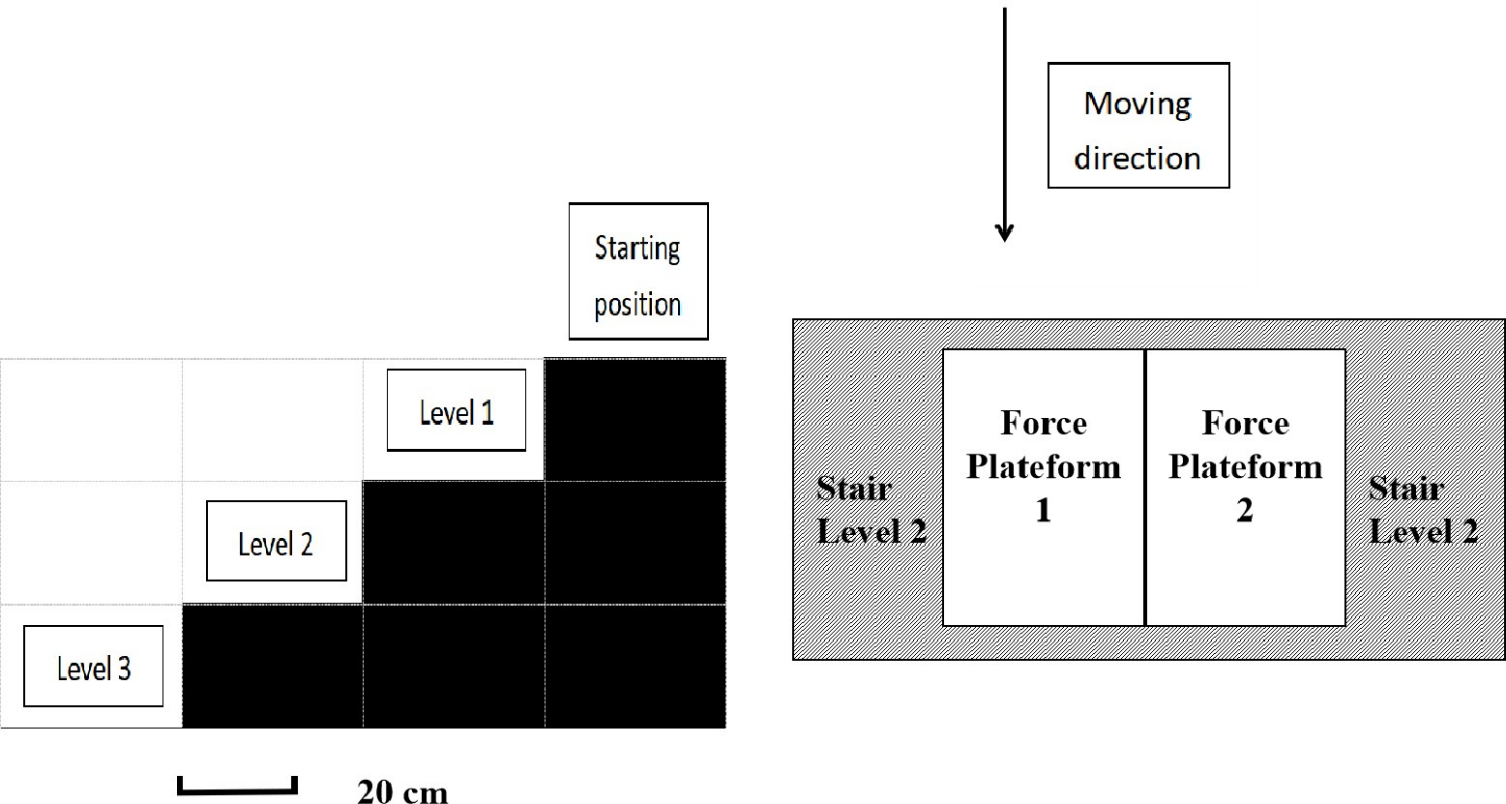
The staircase design (left, side view), and the force platform placement (right, top view).

Body mass, height were measured. The anthropometrics of the segments (including segmental lengths, widths, and circumferences) for both sides of lower extremities were measured. Twenty-nine reflective markers were placed on the anatomical landmarks of the trunk, pelvis, and both lower extremity. All anthropometric measurements and marker placements were performed by the same researcher. A static trial was collected with the subject standing in anatomical position to estimate joint center locations by the markers on the joints. All the participants performed five trials of stair descent on the three-step staircase (height of each level: 19 cm, Fig 1) for each of 6 conditions with their comfortable speed. The order for the conditions in the test was randomized, all 6 conditions were:

1. condition 1: step-over-step without external load;
2. condition 2: step-over-step with 10% of body weight (BW) at the right hand side;
3. condition 3: step-over-step with 10% BW at the left hand side;
4. condition 4: step-by-step without external load;
5. condition 5: step-by-step with 10% BW at the right hand side;
6. condition 6: step-by-step with 10% BW at the left hand side.

For the step-over-step (SOS) condition, the measured side was the right leg (dominant side for all participants). Each trial was started with the left foot (the first step) which would land on the upper level of the staircase (level 1), and the 2^nd^ step was taken with right foot which would land on the force platform on the next lower level of the staircase. For step-by-step (SBS) conditions, participants started each step with right foot (defined as the leading led), the left foot (defined as the trailing leg) followed and landed on the same level of the staircase for each step. Force platforms were placed on the second level of the staircase to measure both legs. Two AMTI force platforms (1600 Hz, AMTI, Watertown, MA) were placed on the lower stairs to measure ground reaction forces. Motion data were collected using an 8-camera system (160 Hz, Vicon MX, Centennial, CO).

Ground reaction forces and motion data were filtered using a low-pass Butterworth filter with a cutoff frequency of 6 Hz [33]. The stance phase cycle for stair descent began at the first contact on the force platform of the measured foot and finished with its toe-off. All gait cycles were normalized into the percentage of the stance phase. A rigid body model and obtained segment masses, center of mass locations, and moments of inertia [34] were used with the inverse dynamics procedures to estimate three-dimensional joint moments and reaction forces at the ankle, knee, and hip. Joint moments and reaction forces were calculated in the global coordinate system and then transformed into the coordinate system of the proximal segment at each joint. All the procedures were performed by the Matlab programs.

The three dimensional segmental angles were used in an individually scaled musculoskeletal model [35, 36]. This model estimated the dynamic muscle-tendon length and velocity adjusted maximal muscle forces, muscle moment arms and orientations for 44 lower limb muscles. Static optimization was used to select a set of muscle forces that minimized the sum of the squared muscle stresses [37] and balanced using the sagittal plane hip, knee and ankle moments, frontal plane hip moment and the transverse plane hip and ankle moments. Solutions were also constrained by the maximal dynamic muscle forces estimated with the musculoskeletal model.


$$Min\;\sum_{i=1}^{44}(F_{i}/A_{i}{)}^{2}\;Subject\;to:\;r_{ij}\times F_{i}=M_{j}\;0\leq F_{i}\leq Max\;dynamic\;F_{i}$$

For the ith muscle: F_i_ is the estimated muscle force, A_i_ is the cross-sectional area, r_ij_ is the moment arm for the jth joint moment, and M_j_ is the jth joint moment.

The 3-dimensional hip joint reaction forces were summed with muscle forces from muscles that crossed the hip joint to obtain hip joint contact forces. The selected muscles were gluteus maximus, gluteus medius, gluteus minimus, biceps femoris (long head), semimembranosus, semitendinosus, quadriceps femoris, rectus femoris, adductor brevis, adductor longus, adductor magnus, gracilis, piriformis, gemelli, tensor fasciae latae, iliacus, psoas, sartorius, pectineus. The 3-dimensional hip joint contact forces were then acting on the femoral head of the femur model. Previous study used this process and compared with the direct measurements (instrumented hip prostheses), which showed an acceptable accuracy about 10–14% difference [38].

The VAKHUM database [39] provided the model (with the geometry and material properties) for the whole femur, which was developed by the clinical CT scans of the femur from a female cadaver (age: 99-yr; mass: 55 kg; height: 1.55 m), and the apparent density was calculated [40]. The finite element model contains 104,945 linear hexahedral elements with 115,835 degrees of freedom (or nodes). The default element edge length is 2.0 mm. Principal stresses, and principal strains will have less than 3% change when increasing element edge length from 2.0 to 3.0 mm, which guaranteed the adequate convergence at the refinement.

The geometry of the model was scaled by the individual thigh length in longitudinal direction, and then scaled by the length·diameter^2^ ∝ body mass [41] in radial direction. The material property was justified by the correlations between Young’s modulus and age. This correlation was justified by the baseline material property from the VAKHUM database [39] and the material property data from other age groups [42]:

$$YM=-0.0391\times Age+19.165,R^{2}=0.8204$$

In which YM stands for the Young’s modulus, Age stands for the age. No significant difference was found between male and female [42].

The basic model contained 286 different linear-elastic material properties, elements were assigned to one of 286 different linear-elastic material properties after justification of the material property. The density-elasticity relationship was based on mechanical testing data of femoral neck [43]:

E = 6850ρ_app_^1.49^, where E is the elastic modulus in MPa, and ρ_app_ is the apparent density in g/cm3; all materials were assigned a Poisson’s ratio of 0.3. The average elastic modulus was 13.5 GPa (ranged from 2.45 to 17.3 GPa) for the cortical bone.

The femur and musculoskeletal models were aligned into a common local coordinate system. 27 femoral muscle insertion locations were mapped to surface nodes of the femur model. The femur was physiologically constrained at the lateral epicondyle (the anterior-posterior direction), center of the patellar groove (all 3 dimensions), and the femoral head contact point (the anterior-posterior, medial-laterial directions) [44]. The 1^st^ principal (tensile) and 3^rd^ principal (compressive) strains at the femur neck were analyzed during both hip joint contact force peaks. The muscle attachment points were excluded in the analysis (FEbio 2.4, University of Utah). All forces (hip joint contact force on the joint center node and attaching muscle forces on the attaching nodes) were applied as point loads [45]. The strain concentrations due to the point loads were removed from further analysis by discarding nodes and elements (load attaching nodes and elements connected with these nodes) in the immediate vicinity of load application [45]. The maximum femoral head deflections and the axial stiffness were tested using FEbio 2.4 to ensure that all the finite element analyses were within a physiologically realistic range.

For the statistical analysis, the peak strains were the averaged strain values from the strain concentration area which contained 6–8 elements in the femoral neck model. The peak strains were analyzed at the two time points during the stance phase that corresponded with the two peak values on the time by hip joint contact force curves for the leg. A two-way repeated-measures MANOVA was used. The independent variables were the gait types (step-over-step vs step-by-step) and the external weight carrying strategies (no weight, 10% body weight at the ipsilateral side, 10% body weight at the contralateral side). The main effect of gait types and external weight carrying strategies as well as a weight strategy by gait type interaction effect on the strains were tested (SPSS, IBM Corp). Univariate ANOVAs were performed if a significant multivariate statistic was found. Pairwise t-tests were used to compare the strains between 2 different gait types with no weight, and among different weight carrying strategies when using the same gait type. If sphericity was violated a Greenhouse-Geisser correction was performed. The alpha level was set at 0.05 for the two-way repeated-measures MANOVA and Univariate ANOVAs. For the pairwise t-tests, due to the multiple comparisons were made, Bonferroni corrections were made on the significance level (gait type comparison: p = 0.0167; weight carrying comparison: p = 0.05).

For the gait type comparison, the percentage of strain differences between two different gait types for both compressive and tensile strains were calculated as:

$$\%DIFF=\frac{Strain_{Greater^{}}-Strain_{Lower}}{Strain_{Greater}}$$

In which %DIFF stands for percentage of strain differences between two different gait types, Strain_Lower_ stands for the condition with lower strains, Strain_Greater_ stands for the condition with greater strains.

For the comparison of weight carrying strategies, the percentage of strain differences was calculated in the similar way above.

## Results

**Fig 2:**
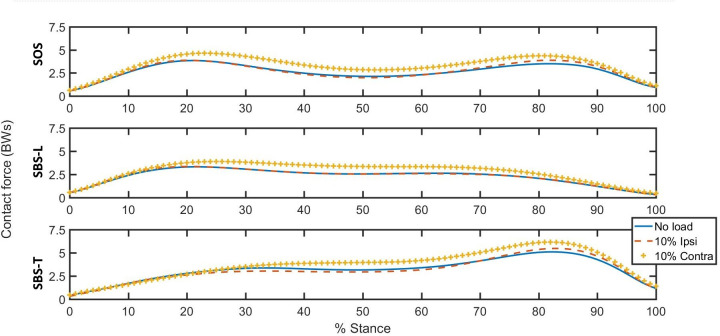
The hip joint contact forces (BW stands for Body Weight) during stair descent for each condition. SOS stands for step-over-step, SBS-L stands for step-by-step with the leading leg, SBS-T stands for step-by-step with the trailing leg. No load stands for no external load, 10% Ipsi stands for 10% of body weight (BW) at right hand side, 10% Contra stands for 10% BW at left hand side.

The maximum femoral head deflections and the axial stiffness in the model were within the physiologically realistic range for all the participants: the femoral head deflections were ranged from 1.7 to 3.3 mm (< 4 mm required), and the axial stiffness was ranged from 1037 to 1230 N/mm [10]. The hip joint contact forces were shown in Fig 2 for each condition.

**Fig 3:**
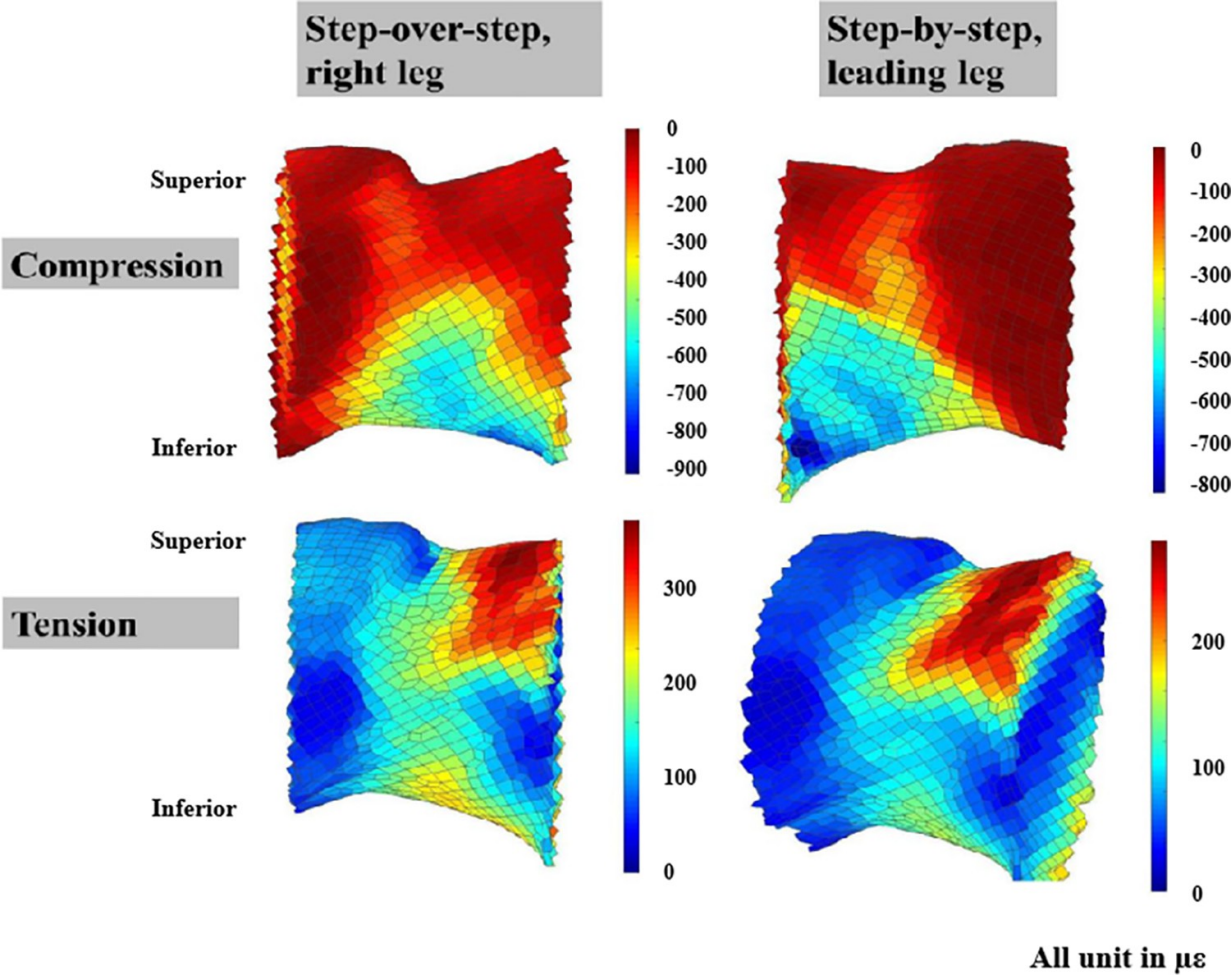
The strain (in με) distribution for the femoral neck during the 1st peak, step-over-step: Posterior view (all); step-by-step: Anterior view (compressive), upper posterior view (tensile). The bottom right femoral neck was set as a different view to get a better view of the strain concentration.

For the SOS condition and the trailing leg for the SBS condition, maximal compressive strains were located at the inferior area of the femoral neck, maximal tensile strains were at the superior area (Fig 3). For the leading leg of the SBS condition, the location of maximal compressive strains shifted anteriorly while maximal tensile strains location shifted posteriorly (Fig 3).

MANOVA results revealed a significant interaction between the gait type (gait) and the external weight carrying strategy (weight) in peak strains (compressive: p<0.001; tensile: p = 0.005). The main effects were also significant for gait type (compressive: p<0.001; tensile: p = 0.011), and weight (compressive: p = 0.005; tensile: p = 0.045). Univariate tests also showed significant results for the effect of gait types (compressive: p = 0.002; tensile: p = 0.002) and weight carrying methods (compressive: p = 0.001; tensile: p = 0.003) on the femoral neck strains.

The gait type effect showed significant differences for both compressive and tensile strains (Table 1). At peak 1, the compressive strains were significantly reduced for the trailing leg (SBS) than the SOS method (p = 0.013, d = 0.69, %DIFF = 12.3%); and at peak 2, these strains were significantly reduced for the leading leg (SBS) than the SOS method (p = 0.012, d = 0.74, %DIFF = 17.4%). No significant difference was found for the rest comparisons at peak 1 (all p>0.04) or peak 2 (all p>0.15).

**Table 1:** Means (SD) of peak strains (in με) at the femoral neck during stair descent without weight carrying, comparing different gait types (significant statistical p-values and effect sizes in bold).

	Compressive		Tensile	
	Peak 1	Peak 2	Peak 1	Peak 2
	No load	No load	No load	No load
Step-over-step (SOS)	-874.1	-736.4	312.5	248.7
	(201.9)	(169.7)	(102.4)	(85.5)
Step- by-step leading (SBS-L)	-820.7	-608.1	289.0	221.1
	(203.0)	(246.1)	(102.4)	(119.4)
Step- by-step trailing (SBS-T)	-766.4	-680.7	325.4	293.8
	(164.3)	(159.3)	(97.8)	(97.5)
SOS v.s. SBS-L	p = 0.041	**p = 0.012**	p = 0.151	p = 0.137
	d = 0.54	**d = 0.74**	d = 0.37	d = 0.38
SBS-L v.s. SBS-T	p = 0.198	p = 0.195	p = 0.048	**p < 0.001**
	d = 0.33	d = 0.35	d = 0.52	**d = 0.76**
SOS v.s. SBS-T	**p = 0.013**	p = 0.151	p = 0.483	p = 0.031
	**d = 0.69**	d = 0.35	d = 0.17	d = 0.57

For the tensile strains, greater tensile strains were found for the trailing leg (SBS) than the leading leg (SBS) at peak 2 (p<0.001, d = 0.76, %DIFF = 24.7%). No significant difference was found for the rest comparisons at peak 1 (all p>0.04) or peak 2 (all p>0.03).

The external weight effect showed more significant differences for the compressive strains (Table 2). The compressive strains were greater for contralateral weight carrying than ipsilateral condition for all 3 gait conditions at peak 1: SOS (p<0.001, d = 0.99, %DIFF = 12.0%), the leading leg SBS (p<0.001, d = 1.40, %DIFF = 9.9%), the trailing leg SBS (p<0.001, d = 1.64, %DIFF = 21.1%); at peak 2 the compressive strains were greater for contralateral weight carrying than ipsilateral condition for SOS gait (p<0.001, d = 1.92, %DIFF = 21.0%). The tensile strains were greater for contralateral weight carrying than ipsilateral condition for the trailing leg SBS (p<0.001, d = 1.07, %DIFF = 24.5%) at peak 1.

**Table 2:** Means (SD) of peak strains (in με) at the femoral neck during stair descent with comparison between contralateral and ipsilateral side weight carrying (significant statistical p-values and effect size in bold).

Compressive strains						
	Peak 1			Peak 2		
	Ipsilateral	contralateral	Statistical	Ipsilateral	contralateral	Statistical
Step-over-step (SOS)	-879.4	-999.6	**p < 0.001**	-736.4	-932.5	**p < 0.001**
	(198.6)	(229.2)	**d = 0.99**	(169.7)	(218.6)	**d = 1.92**
Step- by-step leading (SBS-L)	-838.6	-930.8	**p < 0.001**	-585.5	-527.3	p = 0.36
	(203.3)	(200.0)	**d = 1.40**	(321.0)	(308.7)	d = 0.23
Step- by-step trailing (SBS-T)	-705.8	-894.8	**p < 0.001**	-618.4	-714.3	p = 0.076
	(158.4)	(219.0)	**d = 1.64**	(154.2)	(283.2)	d = 0.46
Tensile strains						
	Peak 1			Peak 2		
	Ipsilateral	contralateral	Statistical	Ipsilateral	contralateral	Statistical
Step-over-step (SOS)	336.3	353.4	p = 0.455	279.7	324.0	p = 0.072
	(134.2)	(113.5)	d = 0.19	(114.2)	(105.9)	d = 0.47
Step- by-step leading (SBS-L)	301.6	319.2	p = 0.55	217.1	186.6	p = 0.141
	(102.3)	(88.3)	d = 0.54	(135.0)	(124.0)	d = 0.38
Step- by-step trailing (SBS-T)	316.1	418.8	**p < 0.001**	277.3	323.4	p = 0.053
	(105.0)	(165.6)	**d = 1.07**	(95.2)	(154.5)	d = 0.50

## Discussion

Stair descent could cause more concerns of femoral neck fractures due to greater stress/strain and increased femoral neck pain or pain related fall risks than ascent [10–15, 24, 25]. Especially for older population with osteoprosis issues, reducing the femoral neck load could be helpful to reduce hip/femoral pain and pain related fall risks. This study tested the effect of different gait types and external load carrying strategies on the femoral neck load during stair descent. In this study, finite element analysis with the human femur model was used in the analysis. Estimated muscular forces and constrained points were provided, and the femur model was scaled according to individual’s thigh length, body mass, age and gender.

The hypothesis that SBS gait strategy could reduce femoral neck strains was partially supported. For peak 1, switching gait types from SOS to SBS could reduce compressive strains at the femoral neck during stair descent for the trailing leg (SBS); no difference was found for the tensile strains. For peak 2, switching gait types from SOS to SBS could reduce compressive strains at the femoral neck during stair descent for the leading leg (SBS); no difference was found for the tensile strains between SBS and SOS gait strategies. These outcomes indicated that SBS strategy could be effective to reduce compressive strains at the femoral neck for the trailing leg (SBS) during the 1^st^ half of stance but not the 2^nd^ half. For the leading leg (SBS), SBS strategy could reduce compressive strains at the femoral neck during the 2^nd^ half of stance but not the 1^st^ half. No significant influence was shown for the tensile strains. Using the injured leg (or leg with hip pain) in SBS gait strategy (either as the leading or trailing leg) could avoid about 10–20% increase of compressive strain at the inferior surface, the SBS method could be helpful to avoid the risks of hip pain (femoral neck pain) or pain related falls and injuries due to the greater decrease of these compressive strains. Moreover, since prolonged exposure to excessive compressive strains could result in bone loss and deterioration, the decrease of compressive strains could be helpful to preserve the bone density and strength.

Different weight carrying methods could change the femoral neck strains. The assumption that contralateral side weight carrying strategy would increase femoral neck strains was made due to the increase of the moment arm for the carrying weight acting on the femoral neck, which increases the bending moment at the femoral neck. Contralateral side weight could increase compressive strains for both SOS and SBS (both the leading and trailing legs) gait strategies during the 1^st^ half of stance, and SOS gait strategy during the 2^nd^ half of stance (Table 2); most tensile strains were not significantly changed due to different weight carrying strategies (Table 2). During stair descent, if external weight carrying is needed, contralateral carrying strategy should be avoided for the injured leg (or leg with hip pain) since it produces 10–20% more compressive strains on the femoral neck than ipsilateral strategy, which could effectively avoid the risks of hip pain (femoral neck pain) or pain related falls and injuries.

In this study, the principal strains at the femoral neck region during stair descent were lower than those from Anderson’s walking tests [29] but similar with the tests from Edwards [30]. This decrease may partially due to 1) different age groups or population for the subjects (average age for Anderson’s older participants were over 70 yrs), 2) about 25% lower speed (0.84–0.92m/s) during stair descent than walking (1.17–1.25 m/s), 3) the difference in the assigned material property/stiffness.

For this study, a generalized femur model was developed based on a 99-yr old female cadaver. Scaling of the geometry [41] and material property [42] are necessary when this model is used for human subjects with different age and gender. This scaling process may result in the element shape distortion for the model, which may have the influence on the accuracy of the analysis. Moreover, all subjects were recruited from the healthy population. Without lower extremity injuries, muscular activities and gait strategies for the subjects could be different from those who have lower extremity injuries (especially femoral neck fractures), so the outcomes of gait strategy change from this study may not be fully applied to injured population. Future studies could focus on 1) incorporating subject-specific imaging with bone model to provide information on bone geometry or density, which could avoid the element shape distortion by using the generalized model; 2) reducing potential errors (errors from calculation, scaling etc.) for the estimation from the musculoskeletal model and finite element models; 3) the age and gender effects on the femoral strains could provide more information for the fall prevention, more work could focus on this effect.

## Supporting information

S1 File Data set. (XLSX)
